# ASO Author Reflections: AI Unraveling Clinical Versus Nonclinical Effects on Post-hepatectomy LOS

**DOI:** 10.1245/s10434-025-17048-8

**Published:** 2025-02-27

**Authors:** Kristin Putman, Samantha M. Ruff, Allan Tsung

**Affiliations:** Department of Surgery, University of Virginia Health, Charlottesville, VA USA

Hepatectomy is a complex and resource-intensive procedure associated with significant postoperative morbidity and prolonged length of stay (LOS). While enhanced recovery protocols and minimally invasive techniques have improved patient outcomes, LOS remains highly variable.^1^ Historically, LOS assessments predominantly concentrate on clinical factors, often overlooking the substantial impact of less tangible nonclinical elements, such as hospital volume, patient care coordination, and social support systems.^2^ This narrow focus limits effective resource allocation and can misinterpret benchmark results. Bridging this gap requires innovative approaches to better understand the interplay between clinical and nonclinical factors driving LOS variability, paving the way for more targeted and effective interventions.

Putman et al. present a cutting-edge artificial intelligence (AI) framework to dissect LOS variability following hepatectomy by quantifying clinical contributions and inferring nonclinical influences.^3^ Using advanced machine learning models, including random forest (RF) and eXtreme Gradient Boosting (XGBoost), this approach captures intricate, nonlinear relationships among clinical variables, achieving predictive performance that exceeds traditional linear models. These findings reveal that clinical factors explain approximately 75% of LOS variability, while nonclinical factors account for the remaining 25%. These results underscore the multifactorial nature of post-hepatectomy recovery and highlight the critical need to consider both clinical and nonclinical dimensions.

Future research must prioritize the direct measurement of nonclinical factors and their integration into LOS reduction strategies. While isolated nonclinical influences—such as bed availability, hospital staffing levels, and psychosocial support—have been explored in other fields, their collective impact on LOS remains underexamined, particularly in hepatectomy patients.^4^ Expanding research to include system-related and socioeconomic variables will provide a more comprehensive understanding of the drivers behind LOS variability and validate existing findings. This holistic approach has the potential to revolutionize quality benchmarking, enhance predictive models, and enable tailored interventions that optimize recovery and resource utilization. By illuminating the multifaceted contributors to LOS, future studies can generate actionable insights to improve patient care and streamline surgical workflows.
